# A Case Report of Salmonella sp. Endocarditis and Literature Review

**DOI:** 10.7759/cureus.79142

**Published:** 2025-02-17

**Authors:** Hermann Do Rego, Yoann Moeuf, Arshid Azarine, Martin Kloekner, Benoit Pilmis

**Affiliations:** 1 Clinical Microbiology Mobile Team (EMMC), Hospital Paris Saint-Joseph, Paris, FRA; 2 Department of Cardiology, Hospital Paris Saint-Joseph, Paris, FRA; 3 Department of Radiology, Hospital Paris Saint-Joseph, Paris, FRA; 4 Department of Cardiology, Hospital Marie-Lannelongue, Paris, FRA

**Keywords:** anti-infective agents, cardio, cardiothoracic surgery, infective endocarditis, salmonella infection

## Abstract

*Salmonella* sp. is a rare cause of infective endocarditis (IE). We present a case of endocarditis diagnosed in an 80-year-old man who had undergone multiple aortic valve replacements and had a recurrence of *Salmonella* sp. bacteremia with hyperfixation on positron emission tomography (PET) scan and an aortic periprosthetic false aneurysm suggestive of a paravalvular abscess on cardiac scan. Treatment consisted of aortic valve replacement and curative antibiotic therapy with ceftriaxone, and the patient is still alive and asymptomatic on suppressive antibiotics with cotrimoxazole. We also present a review of *Salmonella *sp. IE in the PubMed and Google Scholar databases between 2014 and 2023. A total of 39 patients were included, including one case managed by our team. The median age was 55 years, and the most commonly involved valves were mitral and aortic in 43% and 41% of cases, respectively. Thirty-one percent of patients had prosthetic valve endocarditis*. Salmonella enterica *subsp. *enterica* serovar Enteritidis was the main pathogen in 41% of patients. Surgery was performed in 36% of cases. The most common antibiotic was a third-generation cephalosporin in 67% of cases, and the median duration of treatment was six weeks. Mortality under treatment was 10%. In the case of recurrent *Salmonella* bacteremia, endocarditis must be considered.

## Introduction

*Salmonella* spp. are responsible for a variety of human infections, including typhoid fever and non-typhoidal salmonellosis. Typhoid fever is responsible for 22 million cases per year and 200,000 deaths per year, while the incidence of non-typhoidal salmonellosis is 93.8 million cases per year with 155,000 deaths per year worldwide [1,2]. 

These species can cause simple gastroenteritis or bloodstream infections affecting multiple organs (endovascular infections, osteoarticular infections, dermo-hypodermatitis, urinary tract infections, etc.). They may also be responsible for 30% of bloodstream infections in Africa, but their incidence appears to be low in developed countries [3,4].

Endovascular lesions such as aortitis are typical and responsible for high mortality [5]; however, infective endocarditis (IE) caused by *Salmonella* spp. is a rare disease described only in case reports in the medical literature, and there are still no prospective or retrospective studies on the subject.* *

*Salmonella* sp. is estimated to represent 0.03% of cases of IE, compared with *Staphylococcus aureus* in 31% of cases or oral streptococci in 17% of cases, if we refer to certain international prospective cohorts, in particular the International Collaboration on Endocarditis (ICE) cohort (2000-2005) and the GAMES cohort (2008-2018) [6,7].

The most recent reviews on *Salmonella* endocarditis are the study by Cheng et al., published in 2016, which included 87 patients from 1976 and 2014 [8] and the study by Kitazawa et al., published in 2020, which included 38 patients between 1947 and 2019. Four of them were beyond 2012 [9].

However, these studies followed patients over a long period of time and did not take into account improvements in medical management over time. These include early diagnosis using clinical scores such as the Duke criteria, improvements in echocardiography equipment, the use of computed tomography (CT) scanners to better characterize embolic complications and positron emission tomography (PET) scanners to identify infectious sites or prosthesis infections, and better microbiological identification using molecular biology techniques. Treatment has also been optimized with the identification of better antibiotic regimens and early identification of patients who could benefit from early valve replacement surgery [10]. In addition, new guidelines (European and American) have been published in 2015 and 2023.

The aim of this review is to report the case of a patient who had *Salmonella* endocarditis in our center and then to review the most recent cases in the medical literature.

## Case presentation

We present the case of an 80-year-old patient with a history of IE of the native aortic valve in 1992 but no available microbiological data. He underwent bioprosthetic aortic valve replacement for severe aortic stenosis in 2012. In 2018, he presented with a second episode of IE of the bioprosthetic aortic valve, which was documented as *Enterococcus faecalis*. Following the degeneration of the aortic bioprosthesis, he also underwent a valve-in-valve transcatheter aortic valve implantation (TAVI) in 2019 due to his age. His past medical history includes hemolytic anemia due to cold agglutinin disease, atrial fibrillation on curative anticoagulation for stroke prevention, and localized prostate cancer treated by prostatectomy.

On May 5, 2002, the patient presented with chills and asthenia, without fever or other associated symptoms, with a biological inflammatory syndrome (his C-reactive protein (CRP) level was 61 mg/L). As he had a history of prosthetic heart valves, he was admitted to the cardiology department. Three sets of peripheral blood cultures obtained between May 6 and May 7 and separated by at least two hours were positive for *Salmonella enterica* subsp. *enterica* serovar Dublin. Antibiotic susceptibility testing revealed resistance to amoxicillin, ticarcillin, and piperacillin. A transthoracic echocardiogram (TTE) and cardiac CT scan showed no evidence of IE, but the patient refused to undergo a transesophageal echocardiogram (TEE) because of previous discomfort. A thoracoabdominopelvic CT scan was performed to assess the extent of the infection and identify a portal of entry, but no deep-seated infection was found. After seven days of treatment with ceftriaxone, the clinical course and inflammatory syndrome were favorable. Ten pairs of blood cultures were negative between May 8 and May 16, and the patient was discharged from the hospital.

**Figure 1:**
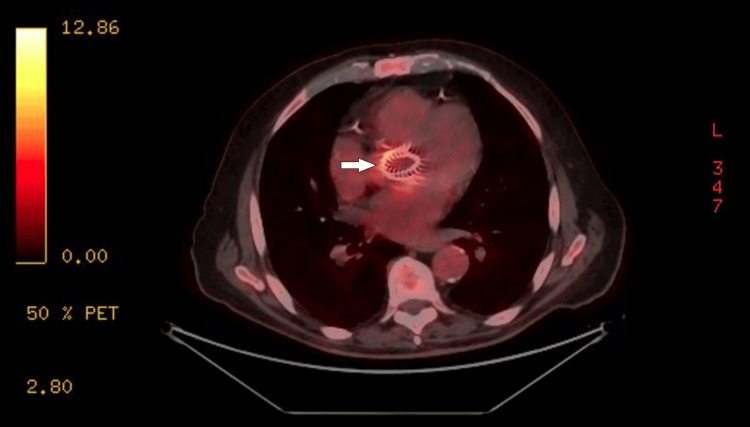
PET scan Circumferential hyperfixation of the aortic bioprosthesis (white arrow) PET: positron emission tomography

On June 2, 2022, the patient presented with fever, chills, rhinorrhea, and exertional dyspnea, without other functional signs, and was referred to the emergency department and then admitted to the internal medicine department where he was diagnosed with biological inflammatory syndrome (his CRP level was 133 mg/L and his nasopharyngeal polymerase chain reaction (PCR) severe acute respiratory syndrome coronavirus 2 (SARS-CoV-2) was negative). Blood cultures obtained on June 3 and June 4 were again positive for *Salmonella* spp. (same antibiogram as serovar as before), and antibiotic treatment with ceftriaxone was started on June 4. Given the recurrence of *Salmonella* bacteremia and the presence of a prosthetic heart valve, a PET scan was performed. It showed circumferential hypermetabolism of the bioprosthesis (SUV max=7) predominantly at the left lateral border without evidence of distant emboli (Figure 1).

**Figure 2:**
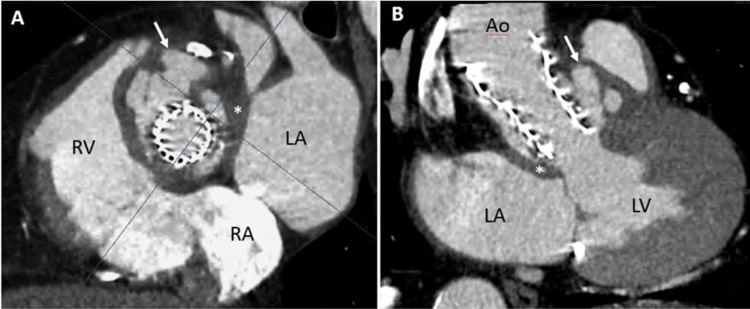
Cardiac CT scan Aortic periprosthetic false aneurysm (white arrow) suggestive of a circulating abscess. (A) Aortic cross-sectional plane showing a multifocal circulating periprosthetic image around the aortic root. (B) Reformatted three-chamber view showing the expansion of the circulating mass during systole CT: computed tomography; RV: right ventricle; RA: right atrium; LA: left atrium; Ao: ascending aorta

The patient was transferred to the cardiac intensive care unit (CICU) for close monitoring due to the development of type 1 atrioventricular block. He was hemodynamically stable. A TTE showed a periprosthetic anechoic image without severe valvular insufficiency, and a cardiac CT scan revealed a 28x19 mm false aneurysm associated with a 5 mm pertussis in the left ventricular (LV) outflow tract (left anterolateral), which in context appeared to be a circulating detergent paravalvular abscess (Figure 2).

Due to the paravalvular abscess, it was decided to proceed with aortic valve replacement with bioprosthesis and abscess debridement on June 15; intraoperative bacteriological samples were negative. The patient improved with apyrexia and reduction of the inflammatory syndrome, with the first negative blood culture on June 8 and subsequent blood cultures until June 25.

**Figure 3:**
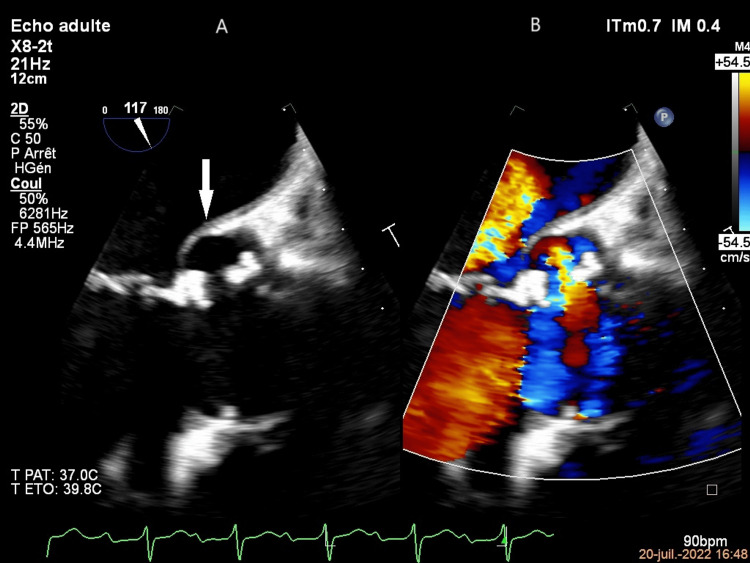
TEE mid-esophageal long-axis view (A) TEE without Doppler color showing the abscess (white arrow). (B) TEE with Doppler color showing the fistulae between the abscess and the aortic root TEE: transesophageal echocardiogram

Antibiotic therapy with ceftriaxone was continued for six weeks, but a TEE performed on July 20 revealed three large false aneurysms around the aortic annulus. All three were at risk of rupture and in communication with the left ventricle, but had no internal echogenic features (Figure 3); the same lesions were seen on the cardiac scan on July 20. Given the high risk of surgical complications, it was decided not to perform a second operation and to continue antibiotic therapy for six weeks. At the end of the curative treatment, the cardiac scan showed the presence of periaortic abscesses of almost the same size as on the July 20 cardiac scan, but there was no increase in fluorodeoxyglucose (FDG) uptake on the PET scan.

Due to the persistence of the false aneurysm and the presence of material, suppressive antibiotic therapy with cotrimoxazole was chosen, given the resistance to amoxicillin and the low risk of acquiring co-drug resistance compared to quinolones. This will be reassessed later depending on the patient's progress.

As *Salmonella* is an *Enterobacteriaceae* that commonly causes gastrointestinal infections, it was decided to perform a colonoscopy, which revealed extensive uncomplicated diverticulosis and multiple subcentimeter polyps. A mucosectomy was performed on four subcentimeter polyps, and pathology revealed tubular adenomas with low-grade dysplasia. According to gastroenterology, this could be the etiology of the bacteremia.

## Discussion

This case study presents the case of an 80-year-old patient with endocarditis, manifested by a recurrence of *Salmonella enterica *subsp. *enterica *serovar Enteritidis and complicated by a circulating abscess around the aorta. The patient required surgical intervention, including valve replacement and drainage of the abscess, followed by prolonged antibiotic therapy. It is unclear whether the recurrence of bacteremia was related to the onset of endocarditis or whether it was a recurrence of the initial bacteremia, but the first hypothesis seems more likely because it was an early recurrence of bacteremia, the PET scan did not show any site of infection other than the perivalvular abscess, and the patient had no functional digestive signs, but one study shows that these are not always present in bloodstream infections with *Salmonella* spp. [4].

In contrast to infectious vasculitis such as aortitis, endocarditis caused by *Salmonella* spp. is a rare disease, with only a limited number of case reports in the medical literature. We performed a literature review of *Salmonella* sp. endocarditis cases published from January 2014 to December 2023 by conducting a bibliographic search in the PubMed and Google Scholar databases using the following keyword: "endocarditis salmonella".

**Figure 4:**
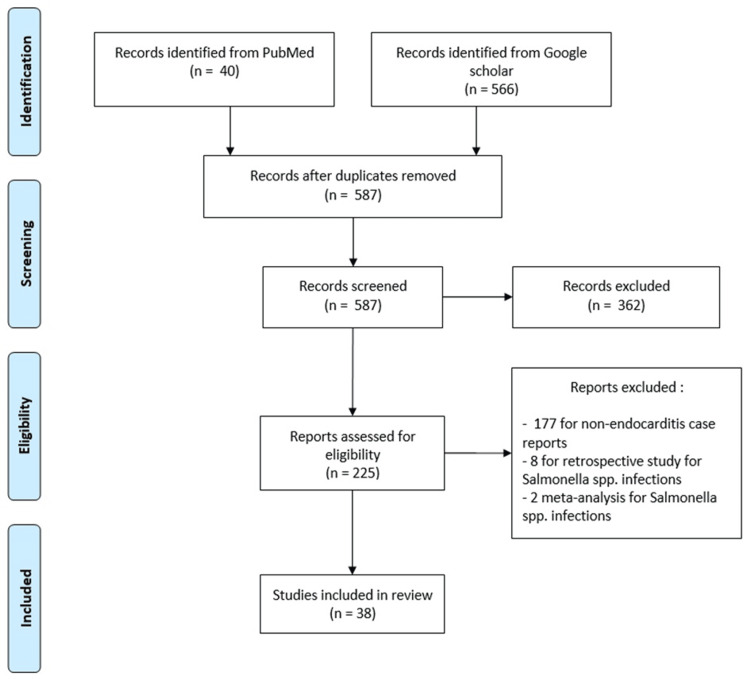
Flowchart

After a search of the PubMed and Google Scholar databases, 38 cases were retained [11-47], and exclusions are detailed in the flowchart (Figure 4). 

In addition to the patient reported in the observation, the review includes 39 patients whose main characteristics are detailed in Table 1.

**Table 1:** Description of the clinical cases Previous bacteremia refers to previous bloodstream infections caused by *Salmonella* spp. Positive blood culture refers to blood cultures positive for *Salmonella* spp. COPD: chronic obstructive pulmonary disease; AF: atrial fibrillation; HIV: human immunodeficiency virus; AIDS: acquired immunodeficiency syndrome; PAH: pulmonary arterial hypertension; TAVI: transcatheter aortic valve implantation; ESBL: extended-spectrum β-lactamase; TTE: transthoracic echocardiogram; PET: positron emission tomography

Studies	Age (years)	Sex	Comorbidities	Germ	Cardiac involvement	Elements of the medical history	Antibiotic/duration	Cardiac surgery performed	Mortality under treatment
Gutiérrez Macías et al. 2014 [11]	82	Man	COPD. AF. Mitral valve prolapse	*Salmonella enterica* subsp. *enterica* serovar Enteritidis	Native mitral	Asthenia, anorexia, fever, inflammatory syndrome. Positive blood cultures and urine cultures. Severe mitral and tricuspid regurgitation. 12 mm mitral vegetation	Ceftriaxone 6 weeks	No	No
Ortiz et al. 2014 [12]	73	Woman	Asthma. Arterial hypertension. Bioprosthetic aortic valve and mitral valve mechanics	*Salmonella enterica* subsp. *enterica* serovar Enteritidis	Mechanical mitral. Biological aortic valve with abscess	Nausea, diarrhea, fever, septic shock, respiratory distress. Positive blood cultures, 11 mm mitral vegetation, aortic valve abscess	Levofloxacin (0.75 mg/kg/d). Then gentamicin (0.6 mg/kg 3 times daily) and aztreonam (20 mg/kg 3 times daily)	No	Yes
Dar et al. 2014 [13]	20	Man	Rheumatic fever	*Salmonella enterica* subsp. *enterica* serovar Paratyphi A	Native mitral	Fever, vomiting, dyspnea, Osler's nodule, subungual hemorrhage, mitral and tricuspid murmur, splenomegaly. Inflammatory syndrome. Positive blood cultures, 5x7 mm mitral vegetation	Ceftriaxone 6 weeks. Chloramphenicol 4 weeks	No	No
Egodage et al. 2015 [14]	40	Man	0	*Salmonella enterica* subsp. *enterica* serovar Paratyphi A	Native mitral	Fever, chills, headache, cough, hepatomegaly. Inflammatory syndrome. Positive blood cultures. 0.6x0.5 cm mitral vegetation	Ceftriaxone (3 g daily)	No	No
Jeyakanth et al. 2015 [15]	66	Woman	Diabetes mellitus. Bronchial asthma. Bioprosthetic aortic valve	*Salmonella enterica* subsp. *enterica* serovar Enteritidis	Aortic bioprosthesis. Native tricuspid	Fever, chills, diarrhea, nausea, hepatomegaly. Inflammatory syndrome, hepatic cytolysis. Positive blood cultures. Aortic vegetation 11×10 mm. Tricuspid valve vegetation 21×12 m. Right ruptured sinus of Valsalva, aneurysm and fistulous communication between the left ventricular outflow track and right atrium	Ceftriaxone (2 g/day) 3 months	No	No
Palangasinghe et al. 2015 [16]	55	Man	Diabetes	*Salmonella enterica* subsp. *enterica* serovar Typhi	Native aortic	Fever, headache, anorexia, nausea, splenomegaly, inflammatory syndrome. Positive blood cultures. Vegetations 5x4 mm	Ceftriaxone 4 weeks. Gentamicin 2 weeks	No	Yes
Blusztein and Strathmore 2016 [17]	47	Man	Heart block. Pacemaker	*Salmonella enterica* subsp. *enterica* serovar Enteritidis	Pacemaker lead	Fever, pacemaker site tenderness. Septic shock. Positive blood cultures	Ceftriaxone	Yes	No
García et al. 2016 [18]	65	Man	Bronchopulmonary cancer on chemotherapy	*Salmonella enterica* subsp. *enterica* serovar Typhimurium. ESBL phenotype	Native aortic	Fever, bilateral coxarthralgia. Inflammatory syndrome. Positive blood cultures and hip joint culture fluid. Addition image of aortic valve on TTE	Piperacillin-tazobactam and then cotrimoxazole and amikacin and then ertapenem 6 weeks of treatment and then ertapenem as suppressive treatment	No	No
Stasev et al. 2017 [19]	25	Woman	Systemic lupus (glomerulonephritis, immune thrombocytopenia, cerebrovascular disease) treated with corticosteroids	*Salmonella enterica* subsp. *enterica* serovar Enteritidis	Native mitral	Fever, chills, dyspnea, alveolar condensation, splenic embolus. Inflammatory syndrome. Positive blood culture. Mitral vegetation 15 mm	Ceftriaxone	Yes	No
Huertas et al. 2017 [20]	50	Man	Chronic hypertension. Diabetes. Myocardial infarction. Heart failure	*Salmonella enterica* subsp. *enterica* serovar Typhi	Mural endocarditis	Fever, diarrhea, abdominal pain, positive blood culture, gangrenous cholecystitis. Septic and cardiogenic shock, 5 mm mural thrombus, ventricular aneurysm	Not described	Yes	No
Laganà et al. 2017 [21]	74	Woman	AF. Arterial hypertension. Psoriasis	*Salmonella enterica* subsp. *enterica* serovar Enteritidis	Mural endocarditis	Fever, emesis, and diarrhea. Ventricular fibrillation with cardiac arrest. Culture of cardiac autopsy	0	0	Yes
Lam et al. 2018 [22]	55	Man	Congenital heart disease. Mechanical aortic and mitral prosthesis. Aortic tube graft. Pacemaker	*Salmonella enterica* subsp. *enterica* serovar Enteritidis	Mechanical mitral and aortic with abscess	Fever, lipothymia. Positive blood culture. Mitral (1.1x0.6 cm) and aortic (0.9x1.1 cm) vegetation. Peri-aortic abscess	Ceftriaxone 6 weeks. Cotrimoxazole (800/160 mg daily) as suppressive treatment	No	No
Piyasiri et al. 2017 [23]	25	Man	0	*Salmonella enterica* subsp. *enterica* serovar Paratyphi A	Native mitral	Fever, headache. Positive blood culture. Mitral vegetation	Ceftriaxone 4 weeks (3 g daily). Then azithromycin (500 mg daily) 1 week	No	No
Robson et al. 2018 [24]	20	Man	0	*Salmonella enterica* subsp. *enterica* serovar Typhi	Native mitral	Previous bacteremia. Fever, asthenia, headache, abdominal pain, inflammatory syndrome. Positive blood and stool cultures. Mitral vegetation	Ceftriaxone 6 weeks (2 g/d). Then ciprofloxacin (500 mg/d) 6 weeks	No	No
Gandhi et al. 2018 [25]	64	Man	AF. Mechanical aortic prosthesis	*Salmonella enterica* subsp. *enterica* serovar Enteritidis	Mechanical aortic	Previous bacteremia. Dyspnea, acute pulmonary edema. Severe aortic insufficiency, periprosthetic leak, proximal aortic dilatation, right aorto-ventricular fistula. Positive blood culture	Ceftriaxone 6 weeks	Yes	No
Dickter et al. 2019 [26]	69	Man	Myelofibrosis complicated by leukemia, allograft with graft-versus-host disease. Pacemaker. Patent foramen ovale with septal implant. Abdominal aortic aneurysm	*Salmonella enterica* subsp. *enterica* serovar Mbandaka	Native mitral	Previous bacteremia. Fever, diarrhea, and abdominal pain. Positive blood and stool cultures. 4 mm mitral vegetation	Ceftriaxone and then amoxicillin/clavulanic acid 3 weeks. Break 1 week. Then cotrimoxazole 1 month. Break 1 week. Then ceftriaxone (2 g/d) 6 weeks	No	No
Youssef et al. 2019 [27]	36	Woman	HIV (17 CD4/m^3^ and viral load at 5.6 log)	*Salmonella enterica* subsp. *enterica* serovar Typhimurium	Eustachian valve	Fever, chills, night sweats, dyspnea, cough, diarrhea. Positive blood culture. Eustachian valve vegetations 6.6x4 mm	Ceftriaxone and then ciprofloxacin 5 weeks	No	No
Hussain and Khalil 2019 [28]	68	Man	Diabetes. Coronary artery disease. Lung cancer undergoing radiotherapy and chemotherapy	*Salmonella* serogroup B	Native aortic	Abdominal pain, inflammatory syndrome. Positive blood culture. Aortic mycotic aneurysm. Vegetations on aortic valve	Ceftriaxone	No	No
Tran et al. 2020 [29]	38	Man	HIV (50 CD4/mm^3^ and viral load at 6.23 log)	*Salmonella enterica* subsp. *enterica* serovar Enteritidis	Native mitral	Diarrhea, recurrent fever, abdominal pain. Splenomegaly, pancytopenia. Cardiogenic shock. Positive blood culture. Nodular mitral vegetation	Not described	No	Not described
Pervin et al. 2020 [30]	62	Woman	Chronic hypertension. Diabetes. Dyslipidemia. Arteritis of the lower limbs. Kidney transplant	*Salmonella enterica* subsp. *enterica* serovar Enteritidis	Native mitral	Fever, confusion, inflammatory syndrome, abdominal pain. Positive blood culture and urine culture positive. Mitral vegetation 0.3x0.3 cm	Ceftriaxone 8 weeks. Amoxicillin as suppressive treatment	No	No
Kitazawa et al. 2020 [9]	76	Man	Gastric cancer. Rheumatic fever	*Salmonella enterica* subsp. *enterica* serovar Enteritidis	Mechanic mitral	Fever, inflammatory syndrome. Left psoas abscess. Hemolytic anemia. Positive blood culture, urine and stool culture. Mitral vegetation 8x6 mm, paravalvular mitral regurgitation	Ciprofloxacin and cefotaxime. Then levofloxacin and ceftriaxone (2 g/day) 14 weeks	Yes	No
Chan et al. 2020 [31]	64	Man	HIV-AIDS stage	*Salmonella *spp. no typhi	Native pulmonary	Fever, dyspnea, chest pain, heart murmur. Bilateral pulmonary embolism, pulmonary infarction, positive blood culture, 10 mm pulmonary vegetation	Ceftriaxone 6 weeks	No	No
Rojas et al. 2020 [32]	48	Man	Acute myeloid leukemia	*Salmonella enterica* subsp. *enterica* serovar Choleraesuis	Native aortic	Fever, chills, dyspnea, heart murmur. Inflammatory syndrome. Positive blood culture. Aortic vegetation 12 mm	Levofloxacin 4 weeks	No	No
Dhayhi et al. 2021 [33]	7	Woman	Sickle cell disease	*Salmonella enterica* subsp. *salamae*	Junction of the right atrium and vena cava	Fever, jaundice, abdominal pain, dyspnea. Hepatosplenomegaly. Inflammatory syndrome. Positive blood culture. Two vegetations of 7 and 8 mm	Cefotaxime and ciprofloxacin 6 weeks with amikacin 2 weeks	No	No
Connolly et al. 2021 [34]	50	Woman	Rheumatic fever. Mitral and aortic mechanical valve replacement. PAH. Hypothyroidism. COPD. Arteritis of the lower limbs	*Salmonella enterica* subsp. *enterica* serovar Enteritidis	Mechanical mitral	Previous diarrhea. Fever, chills, cough, dyspnea, nausea, vomiting, weight loss. Inflammatory syndrome, anemia. Positive blood culture. Long, filamentous, mobile vegetation on mitral valve. Fixation of mitral valve on PET scan	Ceftriaxone and ciprofloxacin 6 weeks. Amoxicillin as suppressive treatment	No	No
Mishra et al. 2021 [35]	50	Man	Chronic alcoholism	*Salmonella* spp. serotype H (type a, type b, type d) and *Salmonella* spp. serotype O, type Vi	Native aortic	Fever, chills, chest pain. Inflammatory syndrome. Positive blood culture. Aortic vegetation 2.5x2.7 mm	Piperacillin/tazobactam then cefepime (4 g daily) and levofloxacin (750 mg daily) 6 weeks	No	No
Rzucidło-Resil et al. 2022 [36]	39	Woman	Barlow's disease	*Salmonella enterica* subsp. *enterica*	Native mitral and aortic	Fever. Inflammatory syndrome. Splenic embolus. Positive blood culture. Mitral and aortic vegetation	Levofloxacin and cotrimoxazole 6 weeks	Yes	No
Alhamadh et al. 2022 [37]	56	Woman	Chronic hypertension. Diabetes. AF. Rheumatic fever. Mechanical mitral and aortic replacement	*Salmonella* spp. groups C and D	Mechanical aortic. Aortic root abscess	Fever, chills, dyspnea, cough, edema of the lower limbs. Inflammatory syndrome, hepatic cytolysis. Positive blood culture. Splenic embolus. Periaortic collection and pseudoaneurysm in the aorta on cardiac scan. Aortic valve and aortic root fixation on PET scan	Ceftriaxone	Yes	No
Allard et al. (2022) [38]	36	Woman	Chronic alcoholism. Addiction to methamphetamine	*Salmonella enterica* subsp. *arizonae*	Native tricuspid	Coma, pleuropneumonia, shock, positive blood and pleural fluid cultures, flaming hemorrhages, tricuspid vegetation 18 mm	Levofloxacin 6 weeks	No	No
Brenneman et al. 2022 [39]	60	Man	AF. Diabetes. Cirrhosis. Pacemaker	*Salmonella enterica* subsp. *enterica* serovar Enteritidis	Pacemaker lead	Previous bacteremia. Fever, chills, abdominal pain. Positive blood culture. Vegetation on 10 mm lead	Ciprofloxacin 1 week. Then ertapenem (1 g/d) 4 weeks	Yes	No
Basyal et al. 2023 [40]	65	Man	*Streptococcus viridans endocarditis*. Mitral valve replacement. Pacemaker	*Salmonella* spp.	Mechanical mitral	Diarrhea, fever, septic shock, renal failure, 15 mm vegetations. Positive blood culture	Ceftriaxone	Yes	No
Kitaya et al. 2023 [41]	66	Man	Chronic hypertension. AF. Chronic renal failure. Aortic valve replacement	*Salmonella enterica* subsp. *enterica* serovar Enteritidis	Mechanical aortic. Aortic ring abscess	Previous bacteremia and gastroenteritis. Fever, para-prosthetic leak, aortic ring abscess. Positive blood culture	Meropenem (3 g/d) 1 week. Ceftriaxone (2 g/d) 6 weeks. Cotrimoxazole (800/160x2/d) as suppressive treatment	Yes	No
Winicki et al. 2023 [42]	57	Man	Polyintoxication (alcohol, tobacco, methamphetamines, cocaine, marijuana)	*Salmonella enterica* subsp. *enterica* serovar Typhimurium	Native aortic	Chills, nausea, diarrhea, acute renal failure, positive blood culture. Small vegetations	Ceftriaxone and levofloxacin 6 weeks	No	No
Zaghdoudi et al. 2023 [43]	9 months	Boy (man)	Operated congenital heart disease (coarctation of the ductus arteriosus and coarctation of the aortic arch). Postoperative endocarditis	*Salmonella enterica* subsp. *enterica* serovar Enteritidis	Pulmonary patch	Fever, tachycardia, systolic murmur, hepatomegaly, inflammatory syndrome. Positive blood culture. Pulmonary patch stenosis with vegetations and pulmonary insufficiency	Amoxicillin (200 mg/kg per day) 6 weeks and gentamicin (5 mg/kg per day) 5 days	No	No
Murray et al. 2023 [44]	26	Man	HIV. Hepatitis C. Polyintoxication (heroin, methamphetamine, cocaine, cannabis)	*Salmonella enterica* subsp. *enterica* serovar Enteritidis	Native mitral and tricuspid	Fever, cough, dyspnea, polypnea, mitral and tricuspid systolic murmurs, inflammatory syndrome, hepatic cytolysis. Positive blood culture, mitral and tricuspid valve perforation, severe valvular regurgitation	Amoxicillin and ceftriaxone 6 weeks	Yes	No
George et al. 2023 [45]	52	Woman	Rheumatic fever. Mitral bioprosthesis. Pacemaker. Heart failure	*Salmonella* spp.	Mitral bioprosthesis	Fever, asthenia, diarrhea. Septic and cardiogenic shock. Positive blood and stool cultures. Mitral valve dehiscence	Not described 6 weeks	Yes	No
van Kruijsbergen et al. 2023 [46]	80	Man	TAVI. COPD. Chronic renal failure	*Salmonella* spp.	Aortic bioprosthesis	Fever, abdominal pain, diarrhea. Inflammatory syndrome, acute on chronic renal failure, moderate aortic insufficiency, TAVI fixation on PET scan. Positive blood culture	Ceftriaxone 6 weeks	Yes	Yes
Zahoor et al. 2023 [47]	25	Man	0	*Salmonella enterica* subsp. *enterica* serovar Typhi. ESBL phenotype	Native aortic	Fever, chills, night sweats, cough, dyspnea, aortic murmur, acute pulmonary edema, aortic vegetations, severe aortic insufficiency, moderate mitral insufficiency. Positive blood culture	Ceftriaxone (2 g/d) and meropenem (2 g/d) 3 weeks	No	No
Our case	80	Man	History of endocarditis. Aortic bioprosthesis. Cold agglutinin disease. AF. Prostate cancer	*Salmonella enterica* subsp. *enterica* serovar Dublin	Aortic mechanical. Circulating aortic abscess	Previous bacteremia. Fever, rhinorrhea, dyspnea. Inflammatory syndrome. Positive blood culture. TAVI fixation on PET scan. Circulating abscess on TTE and coroscanner	Ceftriaxone (2 g/day) 12 weeks. Cotrimoxazole (800/160 mg daily) as suppressive treatment	Yes	No

The median age was 55 years (interquartile range (IQR): 37-65). The majority of patients were male (72%), of whom 23% were immunosuppressed (Table 2).

**Table 2:** Characteristics of the 39 patients Digestive signs include nausea, vomiting, diarrhea, and abdominal pain. Polyvalvular endocarditis means that more than one valve is affected (including the mitral and aortic valves)

Patients' characteristics (n=39)	
Median age, years (interquartile range)	55 (37-65)
Woman, n (%)	11 (28)
Immunodepression, n (%)	9 (23)
Previous bacteremia, n (%)	6 (15)
Digestive signs, n (%)	21 (54)
Prosthetic valve endocarditis, n (%)	12 (31)
Aortic valve, n (%)	16 (41)
Mitral valve, n (%)	17 (43)
Polyvalvular endocarditis, n (%)	5 (13)
Abscess, n (%)	5 (13)
Extra-valvular endocarditis, n (%)	5 (13)
Positive blood culture, n (%)	38 (97)
Embolic complications, n (%)	7 (18)
Germ, n (%)	
*Salmonella enterica* serovar Typhi or Paratyphi	7 (18)
*Salmonella enterica* serovar Enteritidis	16 (41)
*Salmonella enterica* serovar Typhimurium	3 (8)
Type of species not specified	7 (18)
Others	6 (15)
Cardiac surgery	14 (36)
Mortality under treatment	4 (10)

In the medical history, six (15%) patients presented with a first episode of treated bacteremia, classifying the endocarditis as a recurrent infection, and 21 (54%) patients presented with gastrointestinal symptoms (nausea, vomiting, diarrhea, or abdominal pain) before or at the time of endocarditis diagnosis. All blood cultures were positive except for one case report. This was a cardiac autopsy culture because the patient died before blood could be obtained (Table 2).

The most commonly involved valves were the mitral and aortic valves in 41% (16/39) and 43% (17/35) of cases, respectively. Twelve (31%) patients had prosthetic valve endocarditis. Five percent (13/39) of endocarditis cases were complicated by perivalvular abscesses. Embolic complications occurred in 18% of cases (Table 2).

*Salmonella enterica* subsp. *enterica* serovar Enteritidis was the main causative organism in 41% (16/39) of patients, while 18% (7/39) were associated with typhoidal *Salmonella*, either serovar Typhi or Paratyphi (Table 2).

Treatment was medical in 64% (25/39) of cases and medico-surgical in 36% (14/39) (Table 2). The most commonly prescribed antibiotic was a third-generation cephalosporin in 67% (26/39) of cases. Combination therapy was prescribed in 41% of cases. Seventy-five percent of patients (8/12) who had received quinolones were on combination therapy. Nineteen percent (5/26) of patients who had received a third-generation cephalosporin were on combination therapy. The median duration of treatment was six weeks (IQR: 6-6), but in 13 clinical cases, the duration was not described. Suppressive therapy was prescribed in 15% of cases (Table 3). The reasons for cardiac surgery were severe vascular insufficiency in six cases, periprosthetic collection or abscess in three cases, high embolic risk in two cases, pacemaker removal in two cases, and the presence of a pseudoaneurysm in two cases.

**Table 3:** Antibiotic therapy in 39 patients

Patients' antibiotic therapy (n=39)	
Monotherapy (n, %)	23 (59)
Curative antibiotic therapy (n, %)	
Levofloxacin/ciprofloxacin	12 (31)
Ceftriaxone/cefotaxime	26 (67)
Cefepime	1 (2.5)
Meropenem/ertapenem	4 (10)
Piperacillin-tazobactam	2 (5)
Aztreonam	1 (2.5)
Chloramphenicol	1 (2.5)
Cotrimoxazole	4 (10)
Azithromycin	1 (2.5)
Gentamicin/amikacin	5 (13)
Duration of treatment, weeks (interquartile range)	6 (6-6)
Suspensive treatment (n, %)	
Cotrimoxazole	3 (8)
Amoxicillin	2 (5)
Ertapenem	1 (2.5)

Intra-hospital or follow-up mortality was not described but mortality during medical management was 10% (Table 2). The majority of deaths in case reports were related to disease progression and/or surgical complications.

A total of 38 case reports were identified through an exhaustive search of the PubMed and Google Scholar databases between 2014 and 2023, highlighting the rarity of the disease, although there may be non-publication bias.

The population appears to be similar to other cohorts [48,49], with the majority of men having a median age of approximately 55 years and prosthetic valve endocarditis occurring in 31% of cases. As in the reviews by Cheng et al. and Kitazawa et al. [8,9], the main valve involved was the mitral valve in 43% of the cases.

*Salmonella* sp. is a bacterium that causes gastrointestinal infections that may be complicated by bacteremia and secondary foci. However, gastrointestinal symptoms such as diarrhea were found in just over half of the cases, and bacteremia treated before the endocarditis episode was found in 15% of cases, suggesting that endocarditis was responsible for the recurrence.

Typhoidal *Salmonella* was more likely to cause endocarditis in the absence of comorbidities, and of the four patients with no medical history, all were infected with "Typhi" or "Paratyphi" serovars. These serovars are the most virulent in the genus *Salmonella* due to their strict human carriage and the presence of a polysaccharide capsule that protects the bacteria from phagocytosis. Typhoidal *Salmonella* infections could benefit from a more aggressive strategy, such as high-dose antibiotics or dual therapy. However, there are no studies demonstrating this, and in our study, we did not show that typhoidal *Salmonella* caused more deaths, but the number of cases was low (one death, i.e., 14%, compared with two cases of non-typhoidal *Salmonella*, i.e., 12.5%).

The main antibiotics used were third-generation cephalosporins and quinolones, administered as monotherapy in the majority of cases, with the duration of curative treatment ranging from three to 16 weeks, with a median duration of six weeks. The reason for extending treatment beyond six weeks was not justified in all trials, but there did not seem to be an association with complications in the observations in the case reports. With only one death observed with monotherapy (4%) compared with two deaths (16%) with combination therapy, combination therapy did not appear to be more effective than monotherapy. Combination therapy was considered when several antibiotics were used simultaneously (for aminoglycosides for more than five days). Suppressive antibiotic therapy was more likely to be used in patients with immunosuppression or mechanical prostheses. With the exception of the recommended combination therapy, these observations are consistent with European and American guidelines for gram-negative endocarditis. It should be noted that only two cases of extended-spectrum β-lactamase (ESBL) were reported, resulting in treatment with carbapenems.

Due to the retrospective nature of the study and the small number of patients, it is not possible to draw conclusions about the optimal antibiotic regimen and duration of treatment. 

Cardiac surgery was performed in 36% of patients, which is lower than the 50% usually seen in cohorts. With the exception of one case, there were no deaths among patients who underwent valve replacement. We have no explanations or hypotheses to explain the difference in surgery rates in our review, but this underuse of cardiac surgery needs testing in a larger study.

The mortality rate in our study was 10%, which remains lower than that observed in the studies of Cheng et al. [8] and Kitazawa et al. [9], which reported rates of 42.5% and 45%, respectively, when considering cases reported since at least 1946. However, if the period from 2003 to 2014 is considered in the Cheng et al. study, the mortality rate decreases to 13.3%. Furthermore, the EURO-ENDO cohort study [45] reported an in-hospital mortality rate of 17.1%. These differences in mortality are probably due to improvements in the management of endocarditis over time, whether in terms of diagnosis, antimicrobial therapy, or surgery, for example, a European study estimated that early cardiac surgery reduced in-hospital mortality over time [10,50].

The main limitation is that the mortality described in previous reviews [8,9] and our own is based on publishers' reporting, with differences in the definition of mortality, including pre-treatment mortality, end-of-treatment mortality, or mortality at a distance during follow-up. It is also possible that this mortality is underestimated due to possible publication bias, for which the only corrective measure is to establish a prospective cohort over time or to maintain a registry.

Because of the many biases involved in reviewing case reports, we did not perform statistical tests. The observations from this review are purely exploratory, and we hope that they will be used to guide prospective studies or randomized controlled trials, in particular, to determine the optimal duration of treatment, whether to opt for monotherapy or dual therapy, and whether to resort to cardiac surgery.

## Conclusions

Endocarditis caused by *Salmonella* spp. is a rare cardiovascular infection, as this genus is responsible for gastrointestinal disease. If the bacteremia is recurrent or persistent, an echocardiogram (TTE or TEE) should be performed in search of endocarditis, especially if the patient has a prosthetic heart valve. Treatment consists of a course of antibiotics for at least six weeks, including beta-lactam antibiotics (penicillins, cephalosporins) in combination therapy as recommended by guidelines (the European Society of Cardiology and American Heart Association) for gram-negative bacillary endocarditis. Although combination therapy does not appear to improve prognosis in our review, the level of evidence does not allow us to conclude one way or the other. The indication for valve replacement must also be based on guidelines. Compared with previous reviews, our review shows a low mortality rate during treatment and a low need for cardiac surgery. These observations in our review need to be confirmed in a prospective study, as well as the optimal duration of treatment and the use of combination therapy and cardiac surgery.
